# Pathological and Molecular Characterization of H5 Avian Influenza Virus in Poultry Flocks from Egypt over a Ten-Year Period (2009–2019)

**DOI:** 10.3390/ani10061010

**Published:** 2020-06-09

**Authors:** Samah Mosad Mosad, Fatma A. El-Gohary, Hanaa Said Ali, Hanem El-Sharkawy, Ehab Kotb Elmahallawy

**Affiliations:** 1 Department of Virology, Faculty of Veterinary Medicine, Mansoura University, Mansoura 35516, Egypt; dr.sama786@yahoo.com; 2 Department of Hygiene and Zoonoses, Faculty of Veterinary Medicine, Mansoura University, Mansoura 35516, Egypt; dr.fatmagohary@gmail.com; 3 Department of Pathology, Animal Health Research Institute, Mansoura Branch, Mansoura 35516, Egypt; Hanaasaid518@yahoo.com; 4 Department of Poultry and Rabbit Diseases, Faculty of Veterinary Medicine, Kafrelsheikh University, Kafrelsheikh 33511, Egypt; hanem_amin@yahoo.com; 5 Department of Biomedical Sciences, University of León, 24071 León, Spain; 6 Department of Zoonotic Diseases, Faculty of Veterinary Medicine, Sohag University, Sohag 82524, Egypt

**Keywords:** avian influenza, molecular, pathological, characterization, H5, Egypt

## Abstract

**Simple Summary:**

Avian influenza virus (H5) remains one of the challenging zoonotic viruses in Egypt. Our study investigated the occurrence of this virus among chickens from Dakhalia governorate, Egypt over ten years through histopathological examination and molecular characterization of the virus. The molecular characterization was followed by sequencing and phylogenetic analysis of the positive samples. Importantly, we have reported several interesting pathological changes and high occurrence of the H5 avian influenza virus, the phylogenetic analysis revealed that positive samples were aligned with several Egyptian sub clades. Clearly, our study concludes the widespread of the virus among poultry flocks in Egypt and suggests further future research aims to develop an efficient surveillance program with investigation into the effectiveness of the implemented control measures for controlling this disease of public health concern.

**Abstract:**

Avian influenza virus (AIV) remains one of the enzootic zoonotic diseases that challenges the poultry industry in Egypt. In the present study, a total of 500 tissue samples were collected from 100 chicken farms (broilers and layers) suspected to be infected with AIV through the period from 2009 to 2019 from Dakahlia governorate, Egypt. These samples were pooled in 100 working samples and screened for AIV then the positive samples were subjected to histopathological examination combined with real time-polymerase chain reaction (RRT-PCR). RRT-PCR positive samples were also subjected to conventional reverse transcriptase-polymerase chain reaction (RT-PCR) for detection of H5 AIV and some of these resulting positive samples were sequenced for detection of the molecular nature of the studied virus. Interestingly, the histopathological examination revealed necrotic liver with leukocytic infiltration with degenerative changes with necrotic pancreatitis, edema, and intense lymphoid depletion of splenic tissue and hyperplastic tracheal epithelium. Likewise, edema and congested sub mucosal blood vessels and intense bronchial necrosis with hyalinized wall vascular wall and heterophils infiltration were reported. Pneumonic areas with intense leukocytic aggregation mainly and vasculitis of the pulmonary blood vessels were also detected in lung. Collectively, these significant pathological changes in examined tissues cohered with AIV infection. Regarding the molecular characterization, 66 samples were positive for AIV by RRT-PCR and 52 of them were positive for H5 AIV by RT-PCR. The phylogenetic analysis revealed that the H5 viruses identified in this study were aligned with other Egyptian H5N1 AIVs in the Egyptian sub clade 2.2.1, while some of the identified strains were aligned with other Egyptian H5N8 strains in the new Egyptian sub clade 2.3.4.4. Taken together, our present findings emphasize the wide spread of AIV in Egypt and the importance of developing an efficient surveillance and periodical screening program for controlling such disease of public health concern.

## 1. Introduction

Avian influenza (AI) is a highly contagious zoonotic disease caused by the avian influenza virus which is an enveloped single-stranded negative sense RNA virus belonging to the family *Orthomyxoviridae* and genus influenza virus type A [1]. Influenza A viruses encodes at least 10 known proteins; two of these proteins represent the surface glycoprotein spikes; the haemagglutinin (HA) and neuraminidase (NA) which determine virus virulence. Based on the combination of glycoprotein spikes HA and NA found on the virus envelope (18 HA and 11 NA subtypes), influenza A viruses divide into many subtypes [2,3,4]. To our knowledge, all influenza A viruses can infect birds, except subtypes H17N10 and H18N11 which have been found in bats. Based on virus pathogenicity in chickens, avian influenza A viruses are divided into highly pathogenic avian influenza (HPAI) and low pathogenic avian influenza (LPAI). Most identified H5 viruses in poultry and wild birds worldwide are LPAI, but sometimes HPAI have been detected. However, the species barrier can be broken by some AIV subtypes which can infect humans [5,6]. At least eight AIV subtypes have been reported to infect humans, resulting in human influenza pandemics (H1, H2, H3, H5, H6, H7, H9, and H10) [7,8]. Prior to 2013, H5N1, H5N2, H7N2, H7N3, H7N7, H9N2, and H10N7 AIVs were known to cause human infection, then H7N9, H6N1, H10N8, and H5N6 AIVs were also detected in humans [9,10,11,12]. In 2017, H7N9 AIV, which has low pathogenicity in chickens, mutated into a highly pathogenic strain for chickens and caused hundreds of cases of human infections in China [13,14,15,16]. Among others, humans contract the zoonotic strains of the virus through direct contact with infected poultry, implicating the importance of strict hygienic measures to control the disease [10,17]. In accordance with its evolution, the first highly pathogenic H5N1 wave occurred in South East Asia and began with isolation of HPAI H5 from sick geese in Guangdong, China in 1996 [18,19]. Since then, the HPAI H5N1 virus has succeeded to preserve itself in several countries, causing an endemic state with regular outbreaks of the disease [20]. Nowadays, Egypt has been considered the epicenter of the H5N1 outside Asia and hotspot for new subtypes and genotypes evolution [17,21,22]. On the other hand, the HPAI H5N8 was detected for the first time in 2010 in live bird markets in China, then the virus got spread by migratory birds to various areas worldwide including Europe, North America, East Asia, and then in Egypt by December 2016 [23,24,25]. It should be stressed that the Egyptian H5N8 viruses preferentially bound to avian-like receptors rather than human-like receptors [23]. Besides its enormous economic losses, HPAI H5 viruses target public health. The zoonotic impact of the this genotype has been revealed in which 18 humans cases from within Hong Kong were found infected with H5N1 HPAI in 1997, then six of these cases died [26]. The World Health Organization (WHO) reported that 861 humans in 16 countries were infected with H5N1 HPAI and 455 of these cases died by 24 February 2020 [27,28]. Out of these 861 reported human cases, 200 cases were reported from Egypt and 168 of them died [27,28].

Given the above information, the zoonotic importance of the HPAI H5 received worldwide attention and the fear of many international organizations from the risk of continued genetic recombination and production of a pandemic strain of AIV [29,30,31]. Clearly, development of surveillance strategies and investigation into the antigenic shift and phylogenetic analysis in various subtypes of AIV circulating in an endemic area and the continuous screening seem mandatory steps for eradication and control of the disease [32]. In this concern, the present study was undertaken to assess the occurrence of H5 HPAI in commercial chicken flocks in Dakahlia governorate, Egypt through the combined use of pathological identification and molecular characterization of the H5 gene in the studied area.

## 2. Materials and Methods

### 2.1. Ethical Consideration

Ethical approval was obtained from the guidance of Research, Publication, and Ethics of the Faculty of Veterinary Medicine, Mansoura University, Egypt, which complies with all relevant Egyptian legislations. All procedures of collection, handling, preservation, and laboratory analysis of the samples from the birds followed WHO recommended guidelines [33].

### 2.2. Samples and Study Area

A total number of 500 samples (n = 100 liver, n = 100 pancreas, n = 100 spleen, n = 100 trachea, and n = 100 lung samples) were routinely collected between 2009 and 2019 during several outbreaks from 100 poultry farms (72 Broiler and 28 layer farms) in Dakahlia governorate, Egypt. Liver, pancreas, spleen, trachea, and lung samples from each bird were pooled together to get 100 working samples (the rate was 10 working samples per year from 10 farms, which means one sample/farm). These samples were collected with appropriate precautions following WHO recommendations from chickens suspected to be infected with avian influenza virus [33]. Each specimen was separated into two halves: one-half was preserved in 10% formalin and transferred to the Animal Health Research Institute (AHRI), Dokki, Giza, Egypt for histopathology, while the other half was stored at −20 °C until use in RRT-PCR and conventional RT-PCR.

### 2.3. Clinical Signs, Postmortem Lesions, and Histopathological Examination

The clinical signs observed on the poultry flocks and postmortem lesions were recorded. Furthermore, for verification of our results, the conventional RT-PCR H5 positive samples (10% buffered neutral formalin fixed part) were used in histopathological examination. In this concern, liver, pancreas, spleen, trachea, and lung tissues were then processed and embedded in paraffin, sectioned at 7 µm thickness, and stained with hematoxylin and eosin (H and E), according to Perkins and Swayne (2003) [34].

### 2.4. Laboratory Testing

#### 2.4.1. Primers Design

Table 1 shows the primer sets used for both RRT-PCR and conventional RT-PCR, aiming at amplification of both avian influenza virus (M gene) with the control (bird β-actin) and H5 gene, respectively, using some protocols described elsewhere [35,36].

#### 2.4.2. RNA Extraction, cDNA Synthesis, Real Time and Conventional RT-PCR

##### RNA Extraction

Tissue samples (liver, pancreas, spleen, trachea, and lung) from each bird were pooled together and homogenized in sterile mortar and pestle with PBS to get 10% concentration (w/v). These homogenates were then centrifuged and their supernatant fluids were used in viral RNA extraction using the RNeasy mini kit (Qiagen Inc., Valencia, CA, USA) following the manufacturer’s instructions. The concentration and purity of the extracted total RNA was determined by measuring the absorbance ratio at a wavelength of 260 nm over 280 nm using a NanoDrop 2000c spectrophotometer (Thermo Fisher Scientific, Waltham, MA, USA).

##### cDNA Synthesis

Invitrogen™ SuperScript™ III Reverse Transcriptase Kit (Thermo Fisher Scientific, Waltham, MA, USA) was used to convert the extracted viral RNAs to cDNA according to the kit instructions. Reaction mixture (20 µL) was composed in a sterile nuclease free tube on ice by the addition of viral RNA (5 µg), 1.0 μL of specific primer 5′-AGCAAAAGCAGG-3′ (50 mmol/L), 1 µL 10 mM dNTP Mix (10 mM each dATP, dGTP, dCTP, and dTTP at neutral pH), and nucleases free water up to 13 µL. The mixture was then heated to 65 °C for 5 min then incubated on ice for 2 min. Then, 4 µL 5X first-strand buffer, 1 µL 0.1 M DTT, 1 µL RNaseOUT™ (Thermo Scientific, Waltham, MA, USA) Recombinant RNase Inhibitor, and 1 µL of SuperScript™ (Thermo Scientific, Waltham, MA, USA) III RT (200 units/µL) were added. The mixture was then gently mixed by pipetting and incubated at 55 °C for 30 min, then reaction inactivation by heating at 70 °C for 15 min. The cDNA was subsequently stored at −20 °C until use in conventional RT-PCR.

##### Real-Time RT-PCR

Universal avian endogenous RRT-PCR with control (bird β-actin) was used to detect influenza A virus as previously described elsewhere [36]. RRT-PCR was used for the co-amplification of both the influenza M gene and bird β-Actin gene using Luna^®^ Universal One-Step RT-qPCR kit (New England BioLabs), according to the manufacturer’s instructions. The 20.0 μL reaction mixture composed of 10 μL Luna Universal One-Step reaction mix (2X), 1 μL Luna Warm Start RT enzyme mix (20X), and 0.8 µL of each primer (M + 25, M − 124, β + 632, and β − 747, Table 1). Taqman probes were used at final concentration of 250 nM (M + 64 FB and β + 696 FB, Table 1), viral RNA (1 µg), and nuclease-free water was added up to 20 µL. The Applied Biosystems Step One Plus™ real-time PCR instrument was programmed as follows: initial denaturation at 95 °C for 10 min, and 40 amplification cycles of 95 °C for 15 s, and 60 °C for 1 min. Fluorescent signals were obtained once per cycle upon the completion of the extension step.

##### Conventional RT-PCR

RRT-PCR influenza A positive samples were used for amplification of the H5 gene in conventional RT-PCR reaction. The 20 μL reaction mixture composed of 10 μL 2X Dream Taq Green PCR Master Mix (Thermo Scientific, Waltham, MA, USA), 1 µL of each primer for H5 gene (H5-F and H5-R), 2 μL of cDNA, and nuclease free water to a final volume of 20 μL. Amplification was performed in the thermal cycler (Biometra T-Gradient, Goettingen, Germany) as follows: A single cycle of initial denaturation at 95 °C for 3 min, then 40 cycles of denaturation at 95 °C for 30 s, annealing at 54 °C for 30 s, and extension at 72 °C for 40 s. After the last cycle, the reaction was completed by a final extension at 72 °C for 7 min. The PCR products were electrophoresed on 1.5% agarose gel in Tris-acetate-EDTA buffer and 100 bp DNA ladder was used as a molecular weight marker.

#### 2.4.3. Sequencing, GenBank Accession Numbers, and Phylogenetic Analysis of the Selected Samples

In this step, ten samples were selected from H5 gene positive samples (one sample from each collection time) and their nucleotide sequences were determined to confirm the accuracy of the amplified gene. In this regard, the amplified DNA bands of H5 gene PCR products were excised then purified using the gel purification kit (Qiagen, Valencia, CA, USA). The purified PCR products for the selected samples were submitted to the pathology laboratory, University of Liège (Belgium), for bidirectional sequencing using the same primers used in the conventional RT-PCR. The obtained nucleotide sequences were then analyzed and compared versus the other deposited AIV sequences from GenBank (https://www.ncbi.nlm.nih.gov/). MEGA X software was used for sequence analysis and alignments [37]. The nucleotide sequences of the AIV H5 gene fragment from ten selected samples were then deposited in GenBank (NCBI) with accession numbers from MN818673 to MN818682.

## 3. Results

### 3.1. Clinical Signs and Postmortem Lesions

**Figure 1:**
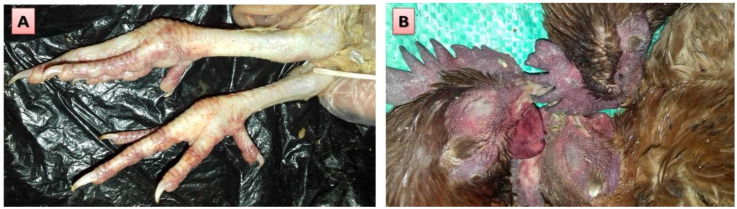
Clinical signs of H5 Avian Influenza Virus (AIV) in tested chickens. (**A**) Cyanosis of legs and (**B**) cyanosis of comb and wattles.

**Figure 2:**
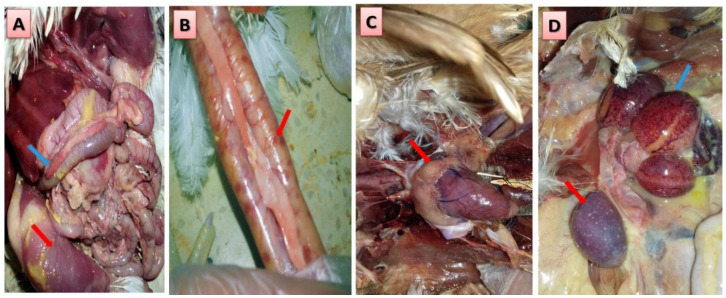
Post mortem changes of H5 AIV in tested chickens. (**A**) Congested muscles (Red arrow) and hemorrhagic pancreas (blue arrow). (**B**) Hemorrhages in intestinal tract. (**C**) Hemorrhages in coronary fat. (**D**) Enlarged, congested, and mottled spleen (red arrow) and hemorrhagic ovarian follicles (blue arrow).

For diagnosis of HPAI, samples were collected from birds suffering from respiratory signs, including coughing, dyspnea, swelling of the infra-orbital sinuses, nasal and ocular discharges, besides high reduction in water and feed intake and diarrhea. Importantly, some birds also showed cyanosis of the legs (Figure 1A), wattles and comb (Figure 1B), and nervous signs with drop in egg production with poor quality eggs in laying birds. The most frequently recorded postmortem lesions included dehydration, and congestion of muscles in some carcasses (Figure 2A) with hemorrhage in gizzard and proventricular mucosae, pancreas (Figure 2A), intestinal tract (Figure 2B), and coronary fat (Figure 2C). Furthermore, spleen was enlarged, congested, and mottled (Figure 2D) with hemorrhagic ovarian follicles (Figure 2D).

### 3.2. Histopathological Examination

**Figure 3:**
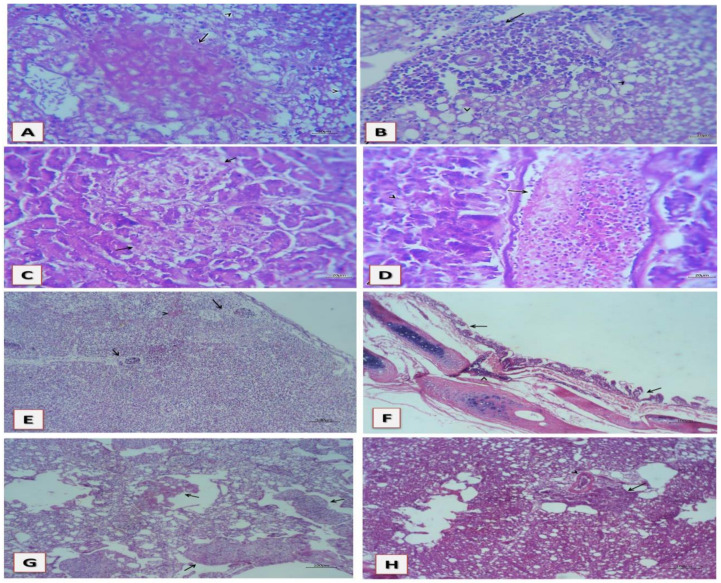
Histopathological changes of H5 AIV in infected chickens: (**A**) liver showing focal necrotic area encircled by a few heterophils and lymphocytes (arrow); (**B**) liver showing portal lymphocytic and heterophilic aggregation (arrow) with necrotic and degenerated hepatic cells (arrow head); (**C**) pancreas showing multiple necrotic areas (arrow); (**D**) pancreas showing minute thrombus admixed with intense heterophils (arrow) besides degenerated and necrotic acini (arrow head); (**E**) spleen showing severe lymphoid depletion of white pulps and minute necrotic areas (arrow head); (**F**) trachea showing hyperplastic mucus glands and mucosal epithelium (arrow) and leukocytosis of sub mucosal B.V.S. (arrow head); (**G**) lung showing disseminated mucus casts containing erythrocytes inside bronchi (arrow); (**H**) lung showing intense perivascular leukocytic aggregation (arrow) and hyalinized vascular wall with endotheliosis (arrow head).

More importantly, the histopathological examination comprised a set of changes that cohere with the infection by HPAI H5. In this concern, the histopathological examination revealed that the liver showed focal necrotic areas randomly distributed with leukocytic infiltration, mainly heterophophils and lymphocytes (Figure 3A). The remaining hepatic parenchyma suffered from degenerative changes, mainly microsteatosis, with intense portal leukocytic aggregations (Figure 3B). Congested blood vessels and hepatic sinusoids with or without recent thrombi were encountered. Furthermore, the pancreatic tissue showed necrotic pancreatitis represented by multiple necrotic areas with or without heterophils infiltration (Figure 3C). Minute recent thrombi disseminated inside some blood vessels admixed with intense heterophils with various degenerative and necrotic changes in the adjacent acini were common (Figure 3D) and sometimes, perivascular hemorrhages could be seen. Likewise, the splenic tissue suffered from intense lymphoid depletion and minute necrotic areas were common (Figure 3E), together with edema with heterophils infiltration could be seen in the capsule, septa, and parenchyma. Trachea showed thickened mucosa by hyperplastic mucus glands and tracheal epithelium besides edema and a few leukocytes, mainly heterophils and lymphocytes, were common (Figure 3F). Sub mucosa had congested blood vessels with or without leukocytosis and edema. Sometimes, mucus exudate could be seen inside the tracheal lumen. The bronchial lumina contained mucus casts mixed with heterophils and erythrocytes beside intense necrosis of bronchial mucosa (Figure 3G). The wall of bronchi had hyalinized vascular wall infiltrated with heterophils with congested blood vessels and edema (Figure 3H). Pneumonic areas represented by intense leukocytic aggregation, mainly heterophils and lymphocytes, beside expanded septae by serofibrinous exudate containing a few erythrocytes were common. Vasculitis of the pulmonary blood vessels with or without endotheliosis and thrombosis could be seen.

### 3.3. Real Time PCR and Conventional PCR

Out of 100 RRT-PCR tested samples, 66 samples (66%) were positive for AIV, while out of these 66 tested samples, 52 samples (78.8%) confirmed to be positive for H5 genes by conventional RT-PCR and the remaining 14 samples (21.2%) were negative.

### 3.4. Sequencing and Phylogenetic Analysis of the AIV H5 Gene Fragment

**Figure 4:**
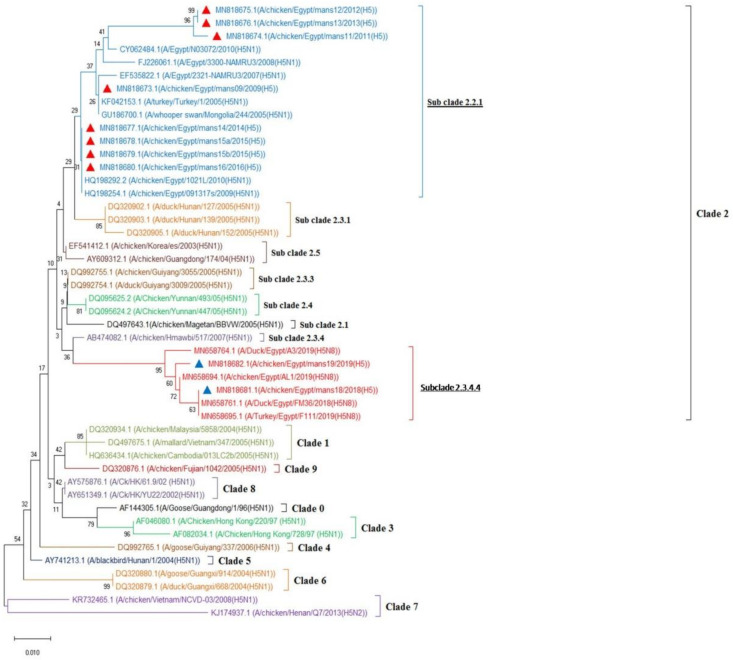
The phylogenetic neighbor-joining tree of 9 AIV clades designed by MEGAX software. The alignment of obtained nucleotides sequences of 10 selected samples shows that the strains mans9, mans11, mans12, mans13, mans14, mans15a, mans15b, and mans16 (red triangles) identified in the present study are closely related to other Egyptian H5N1 viruses as they were aligned in the Egyptian sub clade 2.2.1 containing H5N1 AIVs. The strains mans18 and mans19 (blue triangles) are aligned in the new Egyptian sub clade 2.3.4.4, together with other Egyptian H5N8.

As mentioned above, ten samples were selected (sharp bands) for DNA sequencing (one sample from each collection time) as following mans9/20-Feb-2009, mans11/17-Dec-2011, mans12/3-Dec-2012, mans13/1Mar-2013, mans14/26-Dec-2014, mans15a/7-Jan-2015, mans15b/19-Dec-2015, mans16/2-Mar-2016, mans18/24-Jan-2018, and mans19/2-Feb-2019. The obtained sequences were submitted to GenBank and analyzed in comparison with reference H5 gene sequences in GenBank which are shown in Figure 4. In the present work, the identified H5 AIV showed a close genetic relationship between mans09 strain and both NAMRU/2007 and NAMRU/2008 H5N1 AIV isolated from human in Egypt with identity of 99.48% and 98.97%, respectively. Furthermore, our present data showed 100% identity of mans09 strain with turkey-1/2005 H5N1 strain isolated from turkey poults in turkey and the isolate 244/2005 H5N1 isolated from the whoosper swan from Monogolia. On the other hand, the strains mans11, mans12, and mans13 were closely related to N03072/2010 H5N1 isolated from human in Egypt. In this concern, the identity between mans11 strain and N03072/2010 H5N1 isolated from human in Egypt was 97.42%, while the identity was 96.91% between mans12 and mans13 strains and N03072/2010 H5N1 isolated from human in Egypt. In accordance with mans14, mans15a, mans15b, and mans16, they were closely related to each other to 091317s/2009 H5N1 and 1021L/2010 H5N1 isolated from chickens in Egypt with 100% identity. In addition, the strain mans18 was closely related to FM36/2018 H5N8 AIV isolated from ducks and F111/2019 H5N8 AIV isolated from turkey poults in Egypt with 100% identity for both. In accordance with mans19, it was closely related to AL1/2019 H5N8 AIV isolated from chickens in Egypt with 99.48% identity and A3/2019 H5N8 AIV isolated from ducks in Egypt with 98.97%. Taken together, the H5 strains (mans9, mans11, mans12, mans13, mans14, mans15a, mans15b, and mans16) identified in the present study were aligned in the Egyptian sub clade 2.2.1 containing H5N1 AIVs isolated from human and chickens in Egypt, but the strains mans18 and mans19 were aligned in the new Egyptian sub clade 2.3.4.4, together with other Egyptian H5N8 strains isolated from chicken, duck, and turkey in Egypt (Figure 4).

## 4. Discussion

To the author’s knowledge, Egypt has been considered an endemic country with AIVs since February 2006 [17,21,31,38]. The present findings provide interesting data about the occurrence of the HPAI H5 gene in poultry isolated during several outbreaks from 100 farms in Dakahlia governorate, Egypt over ten years. Interestingly, our study reports several clinical signs, post mortem lesions, and histopathological findings that are compatible with the previous data in the literature in relation to the high pathogenicity of AIV in chickens (HPAI) [39,40,41]. As depicted in our results, the birds experienced some respiratory manifestations and clinical signs including coughing and dyspnea, swelling of the infra-orbital sinuses and nasal discharges, besides some birds showed cyanosis of the legs, wattles, and comb and nervous signs with drop in egg production with poor quality eggs in layers. These current results are in harmony with previous reports where the affected chickens showed similar signs of low feed intake, depression, severe respiratory distress, facial edema with cyanosis of the comb, wattles, shanks, and feet [42,43,44]. However, it should be stressed that some outbreaks did not follow this classical pattern of the gross lesions and some birds may have few or no lesions [45]. In addition to these major reported clinical signs in the present work, the post mortem examination of infected birds showed dehydration and congestion of muscles with hemorrhage in different internal organs, particularly gizzard and proventricular mucosae, pancreas, intestinal tract, and coronary fat. In addition, the spleen was enlarged, congested, and mottled with hemorrhagic ovarian follicles. These reported lesions are consistent with some previous reports with HPAI, where the spleen was found congested, swollen with white necrotic foci, together with petechial hemorrhage in the mucosa of proventriculus and multifocal white necrotic foci in pancreas [40,44,46,47,48].

To the authors’ knowledge, the widespread tissue and endothelial tropism have been considered as marked features and major determinants of the high pathogenicity of HPAI in chickens [41,49,50]. Interestingly, as depicted in Figure 3, the histopathological examination revealed necrosis in liver with infiltration of leukocytes combined with degenerative changes with necrotic pancreatitis, edema, lymphoid depletion of splenic tissue, and hyperplastic tracheal epithelium. Pneumonic areas with intense aggregation of leukocytes and vasculitis of the pulmonary blood vessels were also detected in lung. These present histopathological findings are consistent with several previous pathological data reported with HPAI in the literature [42,44,48,51,52,53]. In these previous studies [42,44,48,51,52,53], trachea and lungs showed congestion, edema, thrombosis, diffuse hemorrhages, necrosis, and sloughing of the epithelial lining of trachea and bronchi, extensive alveolar damage, together with inflammatory cells infiltration including macrophages, lymphocytes, and heterophils. In addition, depletion of the lymphoid follicles and heterophilic cells infiltration was observed in the spleen, together with hemorrhagic foci and necrosis of the periellipsoid lymphocyte sheaths, which is consistent with several previous studies [43,48,51]. Regarding the liver, it showed multiple necrotic foci with leukocytic infiltration, which is in agreement with several previous reports where the liver showed severe congestion, hemorrhage, multifocal hepatic necrosis with fragmented nuclei of the hepatocytes, and infiltration of lymphocytes and macrophages in the periphery of portal triades [48,51,52,54]. Furthermore, the pancreas showed multifocal necrosis with vacuolation of acinar epithelium and infiltration of the mononuclear cells in the parenchyma, as reported elsewhere [48,51,55]. Collectively, our present histopathological data have coincided with several previous findings in lung, spleen, liver, and pancreas HPAI as a result of infection [42,44,48,51,52]. Despite this similarity between the reported pathological changes of AIV H5 infected birds versus previous reports, none of these signs are considered pathognomonic for the disease [40,56,57,58].

Furthermore, the present results were verified by screening of the samples for AIV by RRT-PCR and the resulting positive samples were then subjected to conventional RT-PCR for detection of the H5 gene and were sequenced. In this concern, 66 samples (66%) out of 100 examined samples were positive for AIV using RRT- PCR, while 52 samples (78.8%) out of those 66 samples tested positive by RRT-PCR (confirmed to be positive for the H5 gene by conventional RT-PCR). In fact, several molecular diagnostic methods based on PCR technology have been widely used for AIV detection and genotyping AIV subtypes, such as RT-PCR and RRT-PCR [59,60,61]. Our present results are higher than reported in previous studies in Egypt such as that carried by Elkersh et al. (2019) who reported 30 positive samples out of 89 (30.70%) examined samples for the H5 gene and Kasem et al. (2014) who detected one H5 gene positive sample out of 25 (4%) examined samples [62,63,64]. Among others, migratory and wild birds, variation in the biosecurity measures and hygiene practices of commercial farms, the predominance of live bird trading markets and transportation habits might represent the potential factors that might contribute to this variation of our present results versus the previous studies, favoring the continued endemicity of HPAI H5 [17,63,65,66,67].

Regarding the diversity of AIVs subtypes, several previous studies have described that HPAI H5N1, HPAI H5N8, LPAI H9N2 viruses are the major circulating genotypes among domestic poultry in Egypt [68,69]. Remarkably, the highest number of human cases with the HPAI H5N1 virus in world have been reported in Egypt [28,68]. In fact, the multiple genetic lineages of the genotypes diversity of AIV H5 circulating in the Egyptian poultry, using RT-PCR and partial sequences of HA, offer many advantages for detecting the gene [70]. As shown in Figure 4, the phylogenetic analysis of H5 viruses identified in the present study (mans9, mans11, mans12, mans13, mans14, mans15a, mans15b, and mans16) were aligned in the Egyptian sub clade 2.2.1 containing H5N1 AIVs isolated from human and chickens, but the strains mans18 and mans19 were aligned in the new Egyptian sub clade 2.3.4.4, together with other Egyptian H5N8 strains isolated from ducks, chickens, and turkeys, explaining the continued changes in the molecular nature of H5 AIVs circulating in Egypt. In this concern, the Egyptian A/H5N1 AIV has been reported with at least two distinct genotypes and the virus has mutations associated with increased binding affinity to human receptors, thus posing a public health risk [71]. Clearly, the identity between the present study isolates and human isolates ranging from 96.91% to 99.48% reveals the zoonotic nature of these strains. This close genetic relatedness of our present results clades with the previously reported Egyptian clades and genotypes suggest the existence of single source of infection besides the further spread of the strains that could be related to trade of live birds, and as consequence, this enhances the reassortment activities of influenza subtypes [17,72].

## 5. Conclusions

In conclusion, our study reports interesting histopathological findings, which, together with the molecular data, reveal the high overall prevalence of AIV in the poultry flocks from Dakahlia governorate, Egypt. Clearly, our data provides novel information which requires the attention of local authorities towards the application of more strict hygienic measures and implementation of effective control strategies against this disease of public health importance. Our study also suggests future investigation for the antigenic cartography representation of the Egyptian HPAI (H5) versus LPAI which would be helpful for better understanding the epidemiological pattern of this zoonotic disease in Egypt. Other alternative prophylactic strategies and periodical surveillance regimes may be also essential for proper vaccination and to reduce the risk of infection.

## Figures and Tables

**Table 1:** Detailed primer sets and probes sequences used in RRT-PCR and RT-PCR.

Target	Gene	Primer	Sequence (5′–3′)	Reference
Influenza A virus	M	M + 25	AGATGAGTCTTCTAACCGAGGTCG	[36]
		M − 124	TGCAAAAACATCTTCAAGTCTCTG	
		M + 64FB	FAM-TCAGGCCCCCTCAAAGCCGA-BHQ^TM^1	
Bird Actin	β-Actin	β + 632	CCTCATGAAGATCCTGACAGA	
		β − 747	TCTCCTGCTCYAAYTCCA	
		β + 696FB	FAM-CGTGACATCAAGGAGAAGCTGTG-BHQ^TM^1	
Avian Influenza virus	H5	H5-F	GTACCACCATAGCAATGAGCAG	[35]
		H5-R	AGTCCAGACATCTAGGAATCCGT	
